# Impact of Gut Dysbiosis on Neurohormonal Pathways in Chronic Kidney Disease

**DOI:** 10.3390/diseases7010021

**Published:** 2019-02-13

**Authors:** Nima H. Jazani, Javad Savoj, Michael Lustgarten, Wei Ling Lau, Nosratola D. Vaziri

**Affiliations:** 1 Division of Nephrology, Department of Medicine, University of California-Irvine, Irvine, CA 92697, USA; n_jazani@yahoo.com; 2 Department of Internal Medicine, Riverside Community Hospital, University of California-Riverside School of Medicine, Riverside, CA 92501, USA; javadsavoj@gmail.com; 3 Jean Mayer USDA Human Nutrition Research Center on Aging, Tufts University, Boston, MA 02111, USA; Michael.Lustgarten@tufts.edu

**Keywords:** chronic kidney disease, dysbiosis, gut microbiota, inflammation oxidative stress, prebiotics, probiotics, synbiotics

## Abstract

Chronic kidney disease (CKD) is a worldwide major health problem. Traditional risk factors for CKD are hypertension, obesity, and diabetes mellitus. Recent studies have identified gut dysbiosis as a novel risk factor for the progression CKD and its complications. Dysbiosis can worsen systemic inflammation, which plays an important role in the progression of CKD and its complications such as cardiovascular diseases. In this review, we discuss the beneficial effects of the normal gut microbiota, and then elaborate on how alterations in the biochemical environment of the gastrointestinal tract in CKD can affect gut microbiota. External factors such as dietary restrictions, medications, and dialysis further promote dysbiosis. We discuss the impact of an altered gut microbiota on neuroendocrine pathways such as the hypothalamus–pituitary–adrenal axis, the production of neurotransmitters and neuroactive compounds, tryptophan metabolism, and the cholinergic anti-inflammatory pathway. Finally, therapeutic strategies including diet modification, intestinal alpha-glucosidase inhibitors, prebiotics, probiotics and synbiotics are reviewed.

## 1. Introduction

Chronic kidney disease (CKD) is a major health problem with a high economic burden to healthcare systems all over the world [1,2,3], with a higher global prevalence (11–13%) than diabetes mellitus (8.2%) [3]. It is defined by the presence of a marker of kidney damage such as proteinuria or a reduced estimated glomerular filtration rate (eGFR < 60 mL/min/1.73 m^2^) for at least three months [4]. A remarkable increase in the incidence of CKD has occurred in recent years because of the rising prevalence of hypertension, obesity, and type 2 diabetes mellitus [2]. Other CKD risk factors include smoking [5], nephron loss due to aging and renal senescence [6,7], congenital anomalies of the anatomy and function of the kidney [8], preterm birth and low birthweight [6], and acute kidney injury [6]. The annual mortality rate attributable to CKD is estimated to be approximately one million cases worldwide [1]. Higher rates of CKD prevalence have been reported from developed areas including Europe, USA, Canada, and Australia in comparison with developing countries such as Saharan Africa and India [9].

Conditions that are caused or accelerated by CKD include cardiovascular diseases (CVD, the leading cause of death in CKD), skin abnormalities [10], anemia [11,12,13], cachexia [12,13], sleep disorders [14], psychosocial distress [2], bone disorders fracture [15], hyperphosphatemia and hyperparathyroidism [16,17], hyperkalemia [17], fluid and acid-base disorders [18], and microbial infections [19]. Specific hormonal, inflammatory, nutritional, and metabolic factors may play critical roles in the pathogenesis and progression of CKD. These factors include pro-inflammatory cytokines, such as interleukin (IL)-1, IL-6, tumor necrosis factor (TNF)-α, reduced albumin level, increased C-reactive protein level, reduced growth hormone-insulin like growth factor-1 axis activity, hyperactivation of the renin-angiotensin aldosterone system, and the promotion of insulin resistance [20].

## 2. Gut Microbiota and Symbiotic Benefits

There are 10^13^–10^14^ microbes including 2000–4000 species, both aerobic and anaerobic, residing in the human gastrointestinal (GI) tract, which is collectively referred to as the gut microbiota [21,22]. The gut microbiota is a dynamic and symbiotic ecosystem that is in constant interaction with the host immune system and metabolism [23]. The commensal or symbiotic gut microbiota contains members of three major domains including bacteria (the most abundant), archaea, and eucarya, and density is highest in the colon [21,24,25]. In total, the number of microbial genes is at least 150-fold more than the human genome [26,27]. These microbes have an extensive impact on their host, mainly relating to metabolic pathways for energy harvesting and the production of short-chain fatty acids (SCFAs) and vitamins [26]; it has been suggested that intestinal microbiota should be considered as an additional organ of the body [22]. Seven to nine phyla of bacteria reside in the mammalian GI tract [26,28]. The major bacterial phyla (including genus-level examples) that are present in the gut of healthy humans are *Actinobacteria* (*Bifidobacterium*, *Atopobium*) [29], *Bacteroidetes* (*Bacteroides*, *Prevotella*), *Proteobacteria* (*Proteobacteria*, *Burkholderia*, *Desulfovibrio*) and *Firmicutes* (*Clostridium*, *Eubacterium*, *Roseburia, Ruminococcus*) [21,22,26,30], with *Bacteroidetes* and *Firmicutes* being the dominant phyla [31]. Over 50% of healthy individuals share the same 75 bacterial species, and over 90% of colonic bacteria belong to the *Bacteroidetes* and *Firmicutes* phyla [27].

The diversity of the human gut microbiota varies depending on gender, ethnicity, immune status, nationality, age, diet, geographic location, alcohol and drug consumption, and smoking [32,33,34]. In healthy subjects, the gut microbiota provides several benefits to the host [23]. The gut microbiota protects against pathogens by the inhibition of their colonization via the production of antibiotics and bacteriocins [24,35], facilitates the absorption of complex carbohydrates and produces various nutrients and micronutrients (SCFAs, amino acids such as lysine and threonine, vitamins such as vitamin K6, group B vitamins [23], biotin, and riboflavin [36]) and plays an effective role in element recycling [37]. Furthermore, intestinal microbiota is involved in the development, maturation, and maintenance of GI motility and in shaping the mucosal immune system and intestinal barrier [24,28].

Enterocytes and colonocytes derive 60–70% of their energy from SCFA oxidation [38,39]. SCFAs produced by the gut microbiota can be found in hepatic, portal, and peripheral blood, and influence lipid, glucose, and cholesterol metabolism in various tissues [39]. SCFAs bind and activate specific receptors, such as G-protein coupled receptors FFAR2 (free fatty acid receptor 2, also called GPR43) and FFAR3 (free fatty acid receptor 3, also called GPR41). These receptors are expressed in immune cells, endocrine cells, the GI tract, adipose tissue and the autonomic nervous system, and regulate the host’s energy homeostasis [40]. SCFAs are also involved in immune system activation through neutrophil chemotaxis and the proliferation of regulatory T lymphocytes (Tregs) [41]. Moreover, SCFAs regulate blood pressure through the olfactory receptor 78 (Olfr78) [42] and Gpr41 [43]. Tregs are essential in the maintenance of immunologic self-tolerance [44,45]. The two known types of Tregs are thymus-derived (tTregs) and peripherally-derived (pTregs), which are mainly colon-derived. SCFAs (with butyrate being the most potent) induce the expansion and differentiation of pTregs in the colon and lymphoid tissue [46]. SCFAs additionally have regulatory effects on neutrophils, antigen presenting cells, effector T cells, and natural killer cells [47,48].

**Figure 1:**
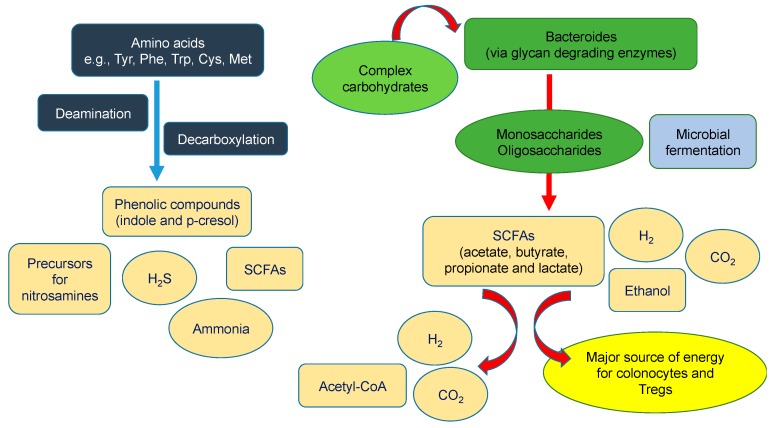
Metabolism of amino acids and carbohydrates by gut microbiota. Complex carbohydrates are converted to monosaccharides and oligosaccharides, and then fermented to hydrogen (H_2_), carbon dioxide (CO_2_), ethanol, and short-chain fatty acids (SCFAs). SCFAs serve as a major source of energy for colonocytes and regulatory T lymphocytes (Tregs), or are converted to acetyl coenzyme-A (Acetyl-CoA), H_2_, and CO_2_. The deamination and decarboxylation of amino acids leads to the formation of ammonia, SCFAs, phenolic compounds, nitrosamines and hydrogen sulfide (H_2_S).

A summary of gut microbiota metabolism resulting in the production of SCFAs is shown in Figure 1.

## 3. Mechanisms of Gut Dysbiosis in CKD

Changes in the composition and function of the microbiota, which is referred to as dysbiosis, has been reported in numerous illnesses including obesity [49], diabetes mellitus [45,50], asthma [45], non-alcoholic fatty liver disease [51], heart failure [45], Parkinson’s disease [52], inflammatory bowel disease [53], CVD [54], cancers [55,56] and CKD [38]. An increased *Firmicutes/Bacteroidetes* ratio has been noted in disease states such as obesity [57], hypertension [58], autism [59] and irritable bowel syndrome [60].

The kidney–gut axis refers to the association between CKD and significant changes in the composition of gut microbiota, the GI environment, and gut epithelial barrier permeability [23,61,62,63,64,65]. Uremic patients show the expansion of specific genera and species of aerobic and anaerobic intestinal bacteria compared to healthy persons [66]. Vaziri et al. showed a significant difference in the abundance of 175 bacterial operational taxonomic units (OTUs) between CKD and control animals, with a significant decrease in *Lactobacillaceae* and *Prevotellaceae*. They also reported significant differences in the frequency of 190 bacterial OTUs between end stage renal disease (ESRD) patients and healthy individuals [23]. It has been shown in CKD and hemodialysis patient cohorts that the number of *Enterobacteriaceae* (*especially* *Enterobacter*, *Klebsiella*, and *Escherichia*), *Enterococci*, and *Clostridium perfringens* were notably higher as compared to healthy controls, but with lower numbers of *Bifidobacterium*, *Lactobacillaceae*, *Bacteroidaceae* and *Prevotellaceae* [67,68,69,70]. Jiang et al. reported that subpopulations of *Roseburia* and *Faecalibacterium prausnitzii* (butyrate-producing species) were significantly reduced in the stool of 65 Chinese patients with CKD in comparison with 20 healthy controls [71]. They proposed that the depletion of butyrate-producing bacteria may play a role in inflammation and CKD progression [71].

Pathways that lead to gut dysbiosis in CKD include: (i) dramatic changes in the biochemical environment of the GI tract induced by an influx of urea, uric acid, oxalate, and other retained waste products from the blood, (ii) diet restrictions, and (iii) medications such as phosphate binders and antibiotics.

### 3.1. Alterations in the GI Tract Biochemical Environment

The influx of urea (the most abundant retained waste product in CKD) and other metabolic toxins into the GI lumen applies a selective pressure favoring the overgrowth of bacteria that produce urease, uricase, indole, and *p*-cresol forming enzymes [45]. Bacterial urease of the gut microbiota hydrolyzes urea and produce ammonium hydroxide, which raises luminal pH and alters the composition of the microbiota [13,35]. Ammonium hydroxide itself is caustic, and leads to the degradation of tight junction barrier proteins [72,73]. Uric acid is the end product of dietary and endogenous purine metabolism in the liver, which is an efficient pathway for the elimination of nitrogen. Oxalic acid is a potentially toxic compound that is not further metabolized in humans, and circulates in its ionized form as oxalate. Under normal conditions, uric acid and oxalate are excreted in the urine; however, the colon plays a major role in the excretion of these compounds in advanced CKD [23,74,75].

### 3.2. Diet

CKD patients are often advised to restrict their intake of fruits, vegetables, and high-fiber products in order to avoid potassium overload. This results in a shortage of indigestible carbohydrates, which are essential nutrients for the gut saccharolytic microbiota, the reduced production of microbial-derived SCFAs, and ultimately decreased nutrients for colonocytes and Treg cells. On the other hand, because of a shortage in carbohydrate resources, the increased metabolism of proteins and other nitrogen-containing substances in the GI tract leads to the production and accumulation of toxic end products. The imbalance between saccharolytic (fermentative) and proteolytic (putrefactive) microbiota is associated with detrimental effects in CKD patients [23,61,76]. CKD patients are also often advised to limit cheese and yogurt consumption because of their high phosphorus content, leading to a deficit of probiotic-rich food sources, which in turn causes more biochemical changes in the GI lumen [45].

### 3.3. Medications

Phosphate binders prescribed to ESRD patients (calcium compounds, sevelamer, lanthanum and iron-based products) bind to phosphorus in the GI tract, and are usually taken with every meal to manage hyperphosphatemia by reducing phosphorus absorption. Alterations in the luminal environment of the GI tract after the long-term consumption of these drugs has been reported [23]. The net benefit/harm balance of iron-containing compounds is controversial; oral iron supplementation to manage chronic anemia in CKD may adversely increase the production of uremic toxins [77]. However, Lau et al. recently reported that ferric citrate (an iron-based phosphate binder) was associated with significant changes in the gut microbiome of CKD rats, including the expansion of a potentially favorable species, *Akkermansia muciniphila*, which has important roles in mucin degradation and gut barrier integrity [78]. CKD patients are often exposed to antibiotics (for example, to treat vascular access infections), which disrupt the gut microbiota via the loss of key taxa and diversity, shifts in metabolic capacity, and expansion of pathogens [23,79].

## 4. Disruption of the Intestinal Epithelial Barrier

Disruption of the intestinal barrier in CKD patients is evidenced by (i) endotoxemia without any evidence of clinical infection [80,81,82], (ii) increased intestinal permeability to polyethylene glycols in CKD humans and animals [83,84], (iii) the detection of GI bacteria in the mesenteric lymph nodes of CKD animals [85], and (iv) histological evidence of chronic inflammation throughout the GI tract (stomach, jejunum, ileum, and colon) [12,86,87]. Urea toxicity, hemodialysis procedure, gut wall edema, inflammation, and oxidative stress are major mechanisms that drive the disintegration of the intestinal barrier [88].

### 4.1. Urea Toxicity

Urease enzyme is expressed by certain microbiota families i.e., Alteromonadaceae, Cellulomonadaceae, Clostridiaceae, Dermabacteraceae, Enterobacteriaceae, Halomonadaceae, Methylococcaceae, Micrococcaceae, Moraxellaceae, Polyangiaceae, Pseudomonadaceae and Xanthomonadaceae [35]. Urease hydrolyzes urea in the gut to form ammonia (NH_3_) which is instantly hydrolyzed to ammonium hydroxide (NH_4_OH). High amounts of ammonia and ammonium hydroxide damage the gut’s epithelial barrier, alter microbiota composition and the luminal biochemical milieu, and result in local and systemic inflammation [35]. A key pathway is the breakdown of epithelial tight junctions via the depletion of occludin, claudin-1 and zona occludens proteins [12,23,72,89]. In CKD rats, decreased expression was at the protein level with mRNA levels remaining constant [12].

### 4.2. Hemodialysis-Associated Disruption of the Intestinal Barrier

Shi et al. examined three patient cohorts including hemodialysis patients, CKD patients not on dialysis, and healthy controls [90], and detected bacterial DNA in the plasma of 27% of hemodialysis patients and 20% of pre-dialysis CKD patients. The majority of bacteria detected in the blood of ESRD patients was also detected in their stool samples, and were not detected in the dialysate solutions [90]. Hemodialysis is thought to exacerbate the CKD-induced injury of the intestinal epithelial barrier [19,23,35,90,91], which was in part due to bowel ischemia from intradialysis and post-dialysis hypotension, and bowel edema due to intradialysis fluid retention, which may be compounded by hypoalbuminemia [13]. Furthermore, systemic anticoagulation, uremic platelet dysfunction, and a high incidence of GI angiodysplasia in these patients can exacerbate intestinal barrier breakdown [19].

### 4.3. Gut Wall Inflammation and Oxidative Stress

As described above, the influx of urea in CKD disrupts the intestinal epithelial barrier. The translocation of endotoxin and bacterial fragments into the sub-epithelial tissue leads to local inflammation via the activation of the resident immune system cells (macrophages, dendritic cells, and T cells), the release of pro-inflammatory cytokines and chemokines, and the infiltration of circulating inflammatory cells [13]. Local production and the release of cytokines such as IFN-γ, TNF-α, IL-12, and IL-1β cause the further disruption of intercellular tight junctions by the induction of endocytosis of claudin-1 and occludin proteins, and by increasing myosin light-chain kinase (MLCK) protein expression and activity [92,93,94,95]. MLCK phosphorylates the myosin regulatory light chain, resulting in the contraction of the actin–myosin ring and increased intercellular permeability [96,97].

## 5. Dysbiosis as a Major Source of Uremic Toxins in CKD

Uremic toxins are classified into three groups: endogenous, exogenous, and microbial-derived [61]. Due to the impaired epithelial barrier in CKD described above, there is a propensity for the translocation of microbe-derived uremic toxins from the GI lumen into the bloodstream. Indoxyl sulfate, *p*-cresyl sulfate and trimethylamine *N*-oxide (TMAO) are the major bacterial-derived toxins [98]. A study of 12 healthy and 24 ESRD individuals revealed a significant expansion of bacterial families possessing urease, uricase, and indole and *p*-cresol forming enzymes, and a reduced number of families possessing SCFA butyrate-forming enzymes [35]. These changes in intestinal microbial metabolism generate uremic toxins, which promote systemic inflammation [35,98]. 

There are currently five different gut-derived uremic toxins that have been associated with CVD and mortality in CKD: indoxyl sulfate, indole-3 acetic acid, *p*-cresyl sulfate, TMAO, and phenylacetylglutamine. Indoxyl sulfate and indole-3 acetic acid are protein-bound uremic toxins generated from bacterial tryptophanase, which is expressed by *Clostridiaceae*, *Enterobacteriaceae* and *Verrucomicrobiaceae* [98]. Tryptophanase converts tryptophan to indolic compounds that are absorbed from the colon, and then sulfated in the liver [99]. Deaminase enzymes produced by *Bacteroides*, *Bifidobacterium*, *Lactobacillus*, *Enterobacter*, and *Clostridium* genera generate phenols by the conversion of tyrosine and phenylalanine to phenyl acetic acid and *p*-cresol, and the latter is conjugated by intestinal microbes to *p*-cresyl sulfate [100]. Trimethylamine is a gut-derived small organic uremic toxin from bacterial metabolism of quaternary amines such as phosphatidylcholine [101] that is absorbed and converted into TMAO by hepatic monooxygenases. Phenylacetylglutamine is another colonic microbial product that is produced from the fermentation of phenylalanine [102]. Indoxyl sulfate, indole-3 acetic acid, and *p*-cresyl sulfate cannot be efficiently removed by conventional hemodialysis, because they are highly bound to albumin [103,104], whereas TMAO and phenylacetylglutamine are water-soluble and dialyzable.

Indoxyl sulfate, *p*-cresyl sulfate, and TMAO are associated with increased cardiovascular morbidity and mortality in CKD patients [45,105,106,107]. In animal models, the oral administration of TMAO has been shown to promote atherosclerosis, and leads to tubulointerstitial fibrosis and progressive kidney dysfunction [45,108,109]. Indoxyl sulfate promotes cardiac fibrosis [110,111] and induces oxidative stress in endothelial cells [35,112]. Indoxyl sulfate’s effects may link gut-derived uremic toxins with the muscle that is wasting observed in CKD [113]. Gene expression of the muscle atrophy markers myostatin and atrogin-1 are increased, while muscle protein synthesis is decreased in the presence of indoxyl sulfate, thereby resulting in decreased skeletal muscle mass [114,115]. Aside from the major known gut-derived toxins, many as yet unidentified toxins in ESRD patients are likely derived from GI microbiota [28].

## 6. The Effect of Dysbiosis on Neuroendocrine Pathways in CKD Patients

**Figure 2:**
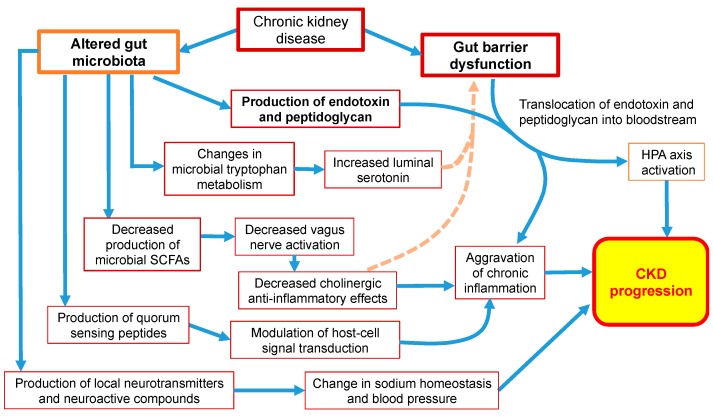
The effect of gut dysbiosis on neuroendocrine pathways in chronic kidney disease. The altered gut microbiota can lead to the activation of the hypothalamic–pituitary–adrenal (HPA) axis; increased serotonin via changes in tryptophan metabolism; the production of neurotransmitters, neuroactive compounds, and quorum sensing peptides; and decreased vagus nerve stimulation via decreased the production of short-chain fatty acids (SCFAs). Ultimately, the HPA axis activation, chronic systemic inflammation, and alterations in sodium and blood pressure hemostasis promote CKD progression.

The reader is directed to the recent paper by Lau et al. that discussed the impact of gut dysbiosis in CKD on the kidney, cardiovascular, bone, adipocytes, and hematologic systems [98]. In this review, we discuss how gut microbiota influence the neuroendocrine system of the host via the hypothalamic–pituitary–adrenal (HPA) axis [116,117], tryptophan metabolism [118], inducing hormone release [119,120], and the production of neurotransmitters, which are neuroactive and hormone-like compounds [121,122,123], and via the vagus nerve (VN) [124]. Alterations in the normal function of the neuroendocrine system due to gut dysbiosis may play a critical role in the establishment and progression of kidney failure (Figure 2).

### 6.1. Hypothalamic-Pituitary-Adrenal (HPA) Axis 

The HPA axis is the major neuroendocrine system of the human body that controls various body processes in response to stress. Due to the bidirectional communication between the gut microbiota and the HPA axis, various disorders of the gut microbiota are associated with HPA axis dysregulation and vice versa. Toxic products of gut microbiota such as endotoxin and peptidoglycan are able to cross the intestinal epithelium barrier, especially under conditions of increased permeability such as CKD, and stimulate the HPA axis either directly or via the activation of the immune system [125]. Overactivation of the HPA axis may result in the progression of CKD in type 2 diabetes mellitus patients, where endogenous hypercortisolism has been associated with HPA axis activation and CKD prevalence [126]. There is a feedback loop whereby activation of the HPA axis alters gut microbiota subpopulations and increases gastrointestinal epithelial barrier permeability [127,128]. 

### 6.2. Induction of Release of Gut Hormones

Larraufie et al. showed that SCFAs (particularly propionate and butyrate) produced by gut microbiota strongly increase the expression and secretion of peptide-YY (PYY) in cultured intestinal cells [129]. PYY is primarily secreted by enteroendocrine cells located in the distal intestine. It plays an important role in the regulation of food intake and insulin secretion. The effect of SCFAs on the expression of this hormone is attributed to the histone deacetylase inhibitory activity of SCFAs and minor contributions of GPR43 [129]. Due to the role of PYY in appetite and energy expenditure, alterations in the expression and secretion of PYY influence the pathophysiology of obesity and hypertension [130] which are important risk factors for CKD [131,132].

### 6.3. Production of Neurotransmitters and Neuroactive Compounds

Gut microbiota produce a wide range of local neurotransmitters and neuroactive compounds [123], including gamma aminobutyric acid (GABA) (produced by *Lactobacillus* and *Bifidobacterium*), serotonin (produced by *Bifidobacterium*, *Streptococcus*, *Escherichia*, *Enterococcus*, *Lactococcus*, and *Lactobacillus*), tryptamine (produced by *Clostridium* and *Ruminococcus*), catecholamine (produced by *Escherichia*, *Bacillus*, *Saccharomyces*, *Lactococcus*, and *Lactobacillus*), and acetylcholine (produced by *Lactobacillus* and *Bacillus*) [122]. Gut microbiota also modulate the production of neurotransmitters through the regulation of the amount and availability of precursors of neuroactive compounds [133,134]. These local neurotransmitters and neuroactive compounds may have critical roles in the regulation of sodium homeostasis and blood pressure, which influence CKD progression [131,135]. 

### 6.4. Tryptophan Metabolism 

Serotonin is a key signaling molecule in both the enteric nervous system and the central nervous system, and is a tryptophan metabolite [136,137,138]. Approximately 95% of the *serotonin* in the body is located in the gut [139]. Therefore, dysbiosis may affect serotonin balance, as microbial tryptophanase activity may limit tryptophan availability to the host [118,140,141]. Bacteria can also synthesize tryptophan through tryptophan synthase [142,143]. *Serotonin* is involved in the control of epithelial *permeability* and the modulation of immune responses [144]. Therefore, changes in the composition and/or activity of the gut microbiota may alter gut permeability through effects on serotonin production or availability. 

### 6.5. Bacterial Hormone-Like Compounds

Bacteria use the quorum sensing system to regulate gene expression and communicate with each other [145]. These communications rely on autoinducer molecules, which are hormone-like compounds that control bacterial physiology and metabolism. Moreover, these molecules can modulate host–cell signal transduction. Some autoinducer molecules interact with host hormones to activate signaling pathways [121,146] and some quorum-sensing peptides (QSP) are able to cross the blood–brain barrier. Although the precise pathways of microbiota-hormonal signaling have not yet been exactly characterized, specific species of gut microbiota have been shown to induce specific changes in hormone levels [121,147]. Changes in QSP patterns may aggravate chronic inflammation which is a risk factor for CKD progression [13,148].

### 6.6. Cholinergic Anti-Inflammatory Pathway

The vagus nerve is the principal component of the parasympathetic nervous system which is composed of 80% afferent and 20% efferent fibers. SCFAs produced by intestinal microbiota may activate vagal chemoreceptors and generate inappropriate responses in the central nervous system (CNS) [149,150,151]. On the other hand, a cholinergic anti-inflammatory pathway through vagus nerve activation may actually reduce peripheral inflammation, inhibit the release of pro-inflammatory cytokines such as TNF-α, and improve intestinal barrier integrity [150,152]. It has been proposed that stimulation of the vagus nerve and activation of the cholinergic anti-inflammatory pathway has an overall protective effect against kidney injury [153]. Heart rate variability is being explored as a marker of gut microbiota-related autonomic dysfunction, as efferent signals from the vagus nerve are predicted to inhibit cytokine production and increase instantaneous heart rate variability [154,155]. 

## 7. Strategies to Attenuate Gut Dysbiosis in CKD

### 7.1. Balanced Diet

Montemurno et al. speculated that the Mediterranean diet—which contains unrefined grains, fruits and vegetables, legumes, nuts, olive oil, fish, and a moderate consumption of red wine—and low amounts of dairy products and red meat may have beneficial gut microbiome effects via providing fiber and antioxidants [156]. On the other hand, a Western diet (rich in animal proteins and fats) stimulates the overgrowth of proteolytic bacteria, which results in dysbiosis, the accumulation of proteolytic-derived uremic toxins such as indoxyl sulfate, and may promote CKD progression [156]. It has been shown that the Mediterranean diet reduces dyslipidemia and protects against lipid peroxidation and inflammation in CKD patients [157]. However, in a cross-sectional study of 276 outpatients who completed a Harvard Food Frequency Questionnaire, the Mediterranean diet score did not correlate with plasma levels of gut-derived uremic toxins including indoxyl sulfate and TMAO [158]. Of note, higher fiber intake in the Dietary Approaches to Stop Hypertension (DASH) diet was associated with a lower incidence of CKD in an elderly Korean population [159].

### 7.2. Prebiotics

Prebiotics are defined as “selectively fermentable ingredients that induce specific modifications in the composition and/or activity of the gut microbiota, which have beneficial effects for the host health” [160]. Prebiotics resist hydrolysis and host absorption and reach the distal GI tract to stimulate the growth and activity of one or a few bacterial species or genera in the colon that are able to ferment these compounds [160]. Some of the prebiotics that are naturally occurring in many fruits, milk and vegetables are fructooligosaccharides, galactooligosaccharides, resistant starch, and lactulose. Beneficial effects are due to the enhanced microbial production of SCFAs and include (i) improved gut barrier integrity and function, (ii) the modulation of anti-inflammatory, antioxidant, and immune system responses, and (iii) the modulation of glucose and lipid metabolism [66,161,162,163].

Our group previously demonstrated that a high resistant starch diet alters the gut milieu, attenuates oxidative stress and inflammation, and improved kidney function in CKD rats. Vaziri et al. compared a low-fiber diet (amylopectin) versus a high fermentable fiber diet (amylose maize resistant starch, HAMRS2) in rats with adenine-induced CKD [44]. The low-fiber diet group showed interstitial fibrosis, inflammation, tubular damage, the activation of NF-κβ, up-regulation of pro-inflammatory, pro-oxidant, and pro-fibrotic molecules, impaired Nrf2 activity, down-regulation of antioxidant enzymes, reduced creatinine clearance, and the disruption of colonic epithelial tight junctions, while the diet high in resistant starch showed significant improvement across all these parameters [44]. In a follow-up report, Vaziri et al. showed that cecal pH was decreased, while *Bacteroidetes/Firmicutes* ratio was increased in HAMRS2-fed rats [164]. Moreover, serum and urine indoxyl sulfate decreased 36% and 66% respectively, and urine *p*-cresol was decreased 47% in HAMRS2-fed rats [164].

### 7.3. Intestinal Alpha-Glycosidase Inhibition

Intestinal alpha-glucosidase inhibitors including acarbose, voglibose, and miglitol are oral glucose-lowering drugs, which act by inhibiting the conversion of carbohydrates into monosaccharides, thus reducing their intestinal absorption and lowering the blood sugar level [165]. These drugs increase the delivery of undigested carbohydrates to colonic microbiota, thereby increasing SCFA production and lowering luminal pH [166]. Two weeks of acarbose supplementation in mice resulted in increased cecal levels of butyrate and total SCFAs in conjunction with increases in *Bacteroidaceae* (genus *Bacteroides*), *Rikenellaceae* (genus *Alistipes*), and *Lachnospiraceae* (genus *Blautia*) [167]. Interestingly, acarbose supplementation in mice increases lifespan [168,169]. In humans, Zhang et al. reported changes in the proportion and diversity of gut microbiota before and after treatment with acarbose in 52 pre-diabetic patients [170]. In a randomized, double-blind, controlled crossover trial, a total of 107 operational taxonomic units (OTUs) were significantly altered after acarbose treatment. Many of the OTUs that were greatly increased with acarbose therapy belonged to SCFA-producing taxa, including *Faecalibacterium*, *Prevotella*, and *Lactobacillus* [170].

The administration of acarbose significantly reduced *p*-cresol amounts in the urine, plasma and feces in a group of individuals with normal kidney function [76], and thus may have benefits in terms of lowering microbial-derived uremic toxins in patients with CKD. 

### 7.4. Probiotics

Probiotics are “live microorganisms which confer health beneficial effects when administered in adequate amounts to the host”, and are administered orally to re-establish the intestinal balance of microbiota. Beneficial effects include pH modulation, the production of SCFAs and anti-bacterial compounds, and the inhibition of pathogenic species [156,171].

In a small randomized double-blind controlled study (16–17 patients per group), Borges et al. investigated the effects of probiotic supplementation on the gut microbiota profile and inflammatory markers in hemodialysis patients. A mixture of *Streptococcus thermophilus*, *Lactobacillus acidophilus* and *Bifidobacterium longum* was administered in a capsule containing 30 billion live bacteria, and participants were prescribed three capsules a day of probiotic or placebo for three months. There was no statistically significant difference in the inflammatory markers and gut profile between two groups [172]. A separate group that studied the Renadyl probiotic formulation in dialysis patients similarly reported no difference in inflammatory markers or quality of life scores [173]. It can be argued that the administration of probiotics without modifying the biochemical environment of the GI tract in CKD would not be able to sustain beneficial effects [28]. 

The classic probiotics that are currently on the market utilize only a small group of organisms. Given that evidence is limited regarding the potential probiotic effects in different disease states, more investigations are needed to find new strains and formulations [174]. *A. muciniphila*, *F. prausnitzii*, *Bacteroides fragilis*, and members of *Clostridia* clusters IV, XIVa, and XVIII have been considered as a “new generation” of probiotics for treatment of the dysbiosis [175]. *F. prausnitzii* is a dominant member in normal gut microbiota, and has beneficial effects, including butyrate production, anti-inflammatory effects by reducing T helper 1 (Th1) and Th17 pro-inflammatory cytokines, and lowering the IL-12 and IFNγ production [175]. As discussed previously, butyrate-producing *Roseburia* and *F. prausnitzii* species are deficient in CKD patients compared with healthy controls [71]. *A. muciniphila*, a mucin-degrading member of gut microbiota, improves endotoxemia-induced inflammation through restoration of the gut barrier [175,176].

*Bacteroides* species are anaerobic commensals in the human GI tract. *B. fragilis* produces polysaccharide A, which is an immunomodulatory molecule that activates Tregs to boost immunologic tolerance [177]. *Eubacterium hallii* is an important anaerobic butyrate and propionate producer that lowers mucosal inflammation and oxidative status, strengthens the epithelial barrier function, and produces SCFAs as an energy source for colonocytes [178,179]. *Clostridium leptum* and *coccoides* are also exceptional inducers of Tregs in the colon [180]. These species deserve further study in newer probiotic formulations.

### 7.5. Synbiotics

Synbiotics contain both probiotics and prebiotics and there have been some beneficial effects reported in CKD patients. Rossi et al. utilized prebiotics including inulin, fructooligosaccharides and galactooligosaccharides with probiotics consisting of nine bacterial strains belonging to *Lactobacillus*, *Bifidobacteria*, and *Streptococcus* genera. Synbiotic treatment significantly decreased serum *p*-cresyl sulfate and improved *Bifidobacterium* counts in stool. A non-significant decrease in serum indoxyl sulfate was also reported [181]. Nakabayashi et al. studied nine hemodialysis patients who received synbiotic treatment with *Lactobacillus casei* and *Bifidobacterium breve*, and galactooligosaccharides as prebiotics. They reported decreased serum *p*-cresol levels in treated patients, but biomarkers of inflammation and oxidative stress were unchanged [182].

Table 1 is a summary of animal and human investigations of prebiotics/probiotics in CKD.

## 8. Summary

In the healthy state, gut microbiota provides several benefits to the host. However, in CKD the heavy influx of urea, uric acid, and oxalic acid compounded with the dietary restrictions and administration of phosphate binders, antibiotics, and oral iron supplements leads to changes in the GI biochemical milieu. Ultimately, there is microbial dysbiosis and disruption of the intestinal epithelial barrier. Dialysis, fluid retention, and hypoalbuminemia also contribute to the increased permeability of the intestinal barrier. Translocation of endotoxin and bacterial-derived uremic toxins into the bloodstream leads to the induction of oxidative stress and inflammation. There is a *bidirectional relationship whereby inflammation* and oxidative stress promote the progression of CKD. Further, gut microbiota affects the brain and neuroendocrine system through several pathways. Prebiotics, new generation probiotics and synbiotics have shown promise in reversing *dysbiosis* in small studies; however, long-term randomized clinical trials are necessary to confirm the efficacy of these compounds in re-establishing symbiotic flora and slowing the progression of CKD.

## Figures and Tables

**Table 1:** Studies that examined prebiotics and/or probiotics in patients or animals with chronic kidney disease.

Species	Dietary Intervention	Study Type	Outcomes	References
**Prebiotics**				
Mice	Short-chain fatty acids (acetate, propionate, and butyrate, pH 7.4 diluted in PBS)	Pilot study	Delayed progression of chronic kidney disease. Improved mitochondrial biogenesis. Reduced local and systemic inflammation, cellular oxidative stress, cell infiltration/activation and apoptosis.	[183]
Rat	Amylose maize resistant starch	Original research study	Attenuation of oxidative stress and inflammation. Delayed progression of chronic kidney disease.	[44]
Rat	High amylose maize-resistant starch type 2 (HAMRS2)	Original research study	Reduction in serum and urine indoxyl sulfate levels. Reduction in urine *p*-cresol level. Improvements in kidney function indexes and amelioration of chronic kidney disease outcomes.	[164]
Human	Gum arabic (highly fermentable fiber)	Clinical trial	Significant decrease in serum urea nitrogen. Significant increase in fecal bacterial mass and fecal nitrogen content.	[184]
Human	Fermentable carbohydrate	Clinical trial	Significant increase in stool nitrogen excretion. Significant decrease in the urinary nitrogen excretion. Unchanged total nitrogen excreted by the two routes. Significant decrease in plasma urea levels.	[185]
Human	Resistant starch	Clinical trial	Significant reduction in plasma indoxyl sulfate. Insignificant reduction in plasma *p*-cresyl sulfate.	[186]
Human	Soluble dietary fiber	Clinical trial	Significant decrease in total cholesterol (TC), low-density lipoprotein (LDL), and TC: LDL ratio. Significant decrease in malondialdehyde, tumor necrosis factor (TNF)-α, interleukin (IL)-6, IL-8, and C-reactive protein levels. No changes in triglycerides, high-density lipoprotein, Cu–Zn superoxide dismutase, and glutathione peroxidase levels.	[187]
Human	Arabinoxylan oligosaccharides	Clinical trial	No significant effect on serum *p*-cresyl sulfate, *p*-cresyl glucuronide, indoxyl sulfate and phenylacetylglutamine. Small, albeit significant decrease in serum trimethylamine *N*-oxide. No change in the urinary excretion of *p*-cresyl sulfate, *p*-cresyl glucuronide, indoxyl sulfate phenylacetylglutamine, and trimethylamine *N*-oxide. No significant change in homeostatic model assessment. No influence on microbiota-derived uremic retention solutes and insulin resistance.	[188]
**Probiotics**				
Rat	Various combinations of *Bacillus pasteurii*, Sporolac, Kibow cocktail, CHR Hansen Cocktail, and Econorm	Pilot study	Improved survival. Reduction in blood urea nitrogen levels. Delayed progression of chronic kidney disease.	[189]
Rat	Soil-borne alkalophilic urease-positive bacterium *Sporosarcina pasteurii*	Pilot study	Reduced blood urea nitrogen levels. Improved survival.	[190]
Rat	*Escherichia coli* DH5 given with urease	Original research study	Reduction of the high plasma urea level to normal	[191]
Dog	VSL#3 supplementation	Original research study	Significant increase in estimated glomerular filtration rate.	[192]
Human	*L. acidophilus*, S. *thermophilus* and *B. longum*	Clinical trial	Significant reduction in blood urea nitrogen levels. Improved quality-of-life scores.	[171]
Human	*L. acidophilus*, *S. thermophilus*, *B. longum*	Clinical trial	Significant reduction of blood urea nitrogen. Moderate reduction in uric acid levels. Insignificant changes in serum creatinine. Improved quality of life scores.	[193]
Human	*B. longum*	Clinical trial	Significant decrease in predialysis serum levels of homocysteine, indoxyl sulfate, and triglycerides.	[194]
Human	*B. longum*	Clinical trial	Reduction in serum indoxyl sulfate.	[195]
Human	*B. longum*	Clinical trial	Delayed progression of chronic kidney disease.	[196]
Human	Lebenin (antibiotic-resistant lactic acid bacteria)	Clinical trial	Reduction in levels of uremic toxins (especially the plasma level of indican).	[67]
Human	*L. acidophilus*	Clinical trial	Reduction of serum dimethylamine and nitrosodimethylamine. Improved nutritional status.	[197]
Human	*Streptococcus thermophilus*, *Lactobacillus acidophilus* and *Bifidobacteria longum*	Clinical trial	Significant increase in serum urea nitrogen. Reduction in fecal pH. No effect on inflammatory markers and gut microbiome profile.	[172]
Human	*Bifobacterium bifidum*, *Bifidobacterium catenulatum*, *Bifidobacterium longum* and *Lactobacillus plantarum*	Clinical trial	Significant reduction in serum TNF-α, IL-5, IL-6, and endotoxin. Significant increase in serum IL-10 levels.	[198]
Human	*S. thermophilus*, *L. acidophilus*, and *B. longum*	Clinical trial	Non-significant improvement in quality-of-life scores. Non-significant reduction of serum indoxyl glucuronide and C-reactive protein.	[173]
Human	*Lactobacillus casei shirota*	Clinical trial	>10% decrease in serum urea concentrations.	[199]
Human	Probiotics	Meta-analysis	Significant reduction in urea level in non-dialysis patients but no change in dialysis patients. No effects on uric acid, C-reactive protein, creatinine, and estimated glomerular filtration rate.	[200]
Human	Probiotics	Meta-analysis	Decrease in *p*-cresyl sulfate. Increase in IL-6. No effects on serum creatinine, blood urine nitrogen, C-reactive protein and hemoglobin levels.	[201]
**Synbiotics**				
Human	Prebiotics; galactooligosaccharides Probiotics: *Lactobacillus casei* strain *Shirota* and *Bifidobacterium breve* strain *Yakult*	Clinical trial	Significant decrease in serum *p*-cresol level. Normalization of bowel habits.	[182]
Human	Prebiotics: inulin high performance, fructo-oligosaccharides, and galactooligosaccharides Probiotics: *Lactobacillus*, *Bifidobacteria*, and *Streptococcus* species	Clinical trial	Significant decrease in serum *p*-cresyl sulfate. Favorable modification of the stool microbiome.	[181]
Human	Prebiotics: Fructooligosaccharides Probiotics: *Lactobacilus casei*, *Lactobacilus acidophilus*, *Lactobacilus bulgarigus, Lactobacilus rhamnosus, Bifidobacterium breve*, *Bifidobacterium longum*, and *Streptococcus thermophilus*	Clinical trial	Significant reduction in blood urea nitrogen levels.	[202]
Human	Prebiotics: Fructooligosaccharides Probiotics: *Streptococcus thermophiles*, *Lactobacillus acidophilus*, *Bifidobacterium longum*	Clinical trial	Significant lowering of the rate of decline in estimated glomerular filtration rate.	[203]
Human	Commercial symbiotic formulation: Probinul neutro	Clinical trial	Significant reduction in total plasma *p*-cresol level.	[204]
Human	Prebiotic and Probiotics	Meta-analysis	Synbiotic interventions significantly increased *Bifidobacterium* in gut microbiota, but had little or no effect on serum urea nitrogen, indoxyl sulfate, and *p*-cresyl sulfate. Prebiotic supplementation may slightly reduce serum urea concentration.	[205]
